# DragonBall: A Suite for Vibrational Spectroscopy with Classical Trajectories

**DOI:** 10.1021/acs.jpca.6c04232

**Published:** 2026-07-20

**Authors:** Giacomo Mandelli, Antonino Famulari, Carlo Cavallotti, Sophya Garashchuk, Giacomo Botti

**Affiliations:** † Department of Chemistry, Materials, and Chemical Engineering “Giulio Natta”, 18981 Politecnico di Milano, Piazza Leonardo da Vinci 32, 20133 Milan, Italy; ‡ Department of Chemistry and Biochemistry, 2629 University of South Carolina, 631 Sumter Street, Columbia, South Carolina 29208, United States of America

## Abstract

One of the most fascinating and key aspects in simulating vibrational spectra is accounting for the anharmonicity of the vibrational motions. The quasi-classical trajectory (QCT) method allows for the computation of fully anharmonic vibrational spectra, using a single trajectory initialized with quantized energy. Unfortunately, the everyday use of QCT requires constant juggling between the different input and output formats of the various quantum chemistry software and the use of different computational and visualization tools among these steps. In this paper, we showcase Dragonball, a modular Python suite that guides the user in each step of the QCT simulation, from guess geometry to final spectrum.

## Graphical abstract

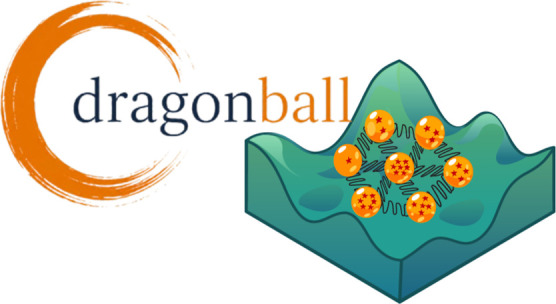

## Introduction

Since the discovery of infrared radiation by William Herschel in 1800,^1,2^ vibrational spectroscopy has continuously evolved into a fundamental pillar of chemical and physical analysis. Initially employed for the structural characterization of simple isolated molecules, the field has grown dramatically in both precision and scope. Today, vibrational spectroscopy extends well beyond the study of gas-phase molecules, and it is routinely employed to investigate complex molecular environments, capturing the subtle interplay between an active molecule and its surrounding bath. From probing noncovalent interactions in microsolvated clusters to monitoring the intricate dynamics of solvent networks or matrix-isolation effects, vibrational spectra are highly sensitive to their environment, offering a direct window into condensed-phase dynamics.^3–5^ Moreover, the knowledge of the vibrational behavior of a system is fundamental in the treatment of the bath degrees of freedom of open quantum system, that often relies on a (vibrational) spectral density formalism.^6–9^

Robust theoretical and computational tools are required to properly interpret these increasingly complex and congested spectral features. The standard double harmonic approximation often serves as a computationally cheap and ubiquitous starting point, but it inherently fails to account for vibrational anharmonicity, overtones, and extensive mode couplings. To recover these missing features, a variety of theoretical tools have been developed, ranging from static postharmonic methods (to mention a few: vibrational perturbation theory—VPT2, vibrational self-consistent field—VSCF, scaled harmonic frequencies, and machine learning scaling)^10–15^ to advanced quantum and semiclassical dynamics.^16–18^

Among these approaches, we have the methods based on classical trajectories. Through the quasi-classical trajectory (QCT) method, anharmonic vibrational frequencies can be extracted directly from a single classical trajectory initialized at the quantized zero-point energy. QCT naturally captures full-dimensional anharmonicity and mode coupling, and when paired with ab initio Born–Oppenheimer molecular dynamics (BOMD), it bypasses the curse of dimensionality associated with the arduous construction of analytical potential energy surfaces.^15,19–21^

As the interest in modeling vibrational anharmonicity grows, there have been commendable attempts to provide the computational chemistry community with dedicated software tools. A notable example is the SEMISOFT web platform recently created by Ceotto and co-workers,^22^ which assists users in computing the vibrational density of states (VDOS) from their own molecular dynamics data. However, despite these advances, a significant practical hurdle remains: to the best of our knowledge, there is currently no comprehensive tool capable of seamlessly guiding the user “from guess geometry to spectrum”. Existing tools primarily focus on the final spectral analysis (providing only visualization and the Fourier Transform tool), leaving researchers with the tedious, manual, and highly error-prone tasks of setting up the ab initio calculations. Preparing the quantized initial conditions to ensure the trajectory runs on the correct phase-space torus, extracting equilibrium Hessians, and juggling the complex I/O formats of various molecular dynamics engines remain significant bottlenecks in the workflow.

Faced with these practical hurdles, one might naturally ask: Do we really need to search for the spheres of the Shenron dragon? To answer this question and bridge the aforementioned gap, we introduce the Dragonball suite, an automated, open-source Python toolbox specifically designed for vibrational spectroscopy with classical trajectories. Designed to alleviate the burdensome I/O and data-handling requirements, Dragonball interfaces directly with standard quantum chemistry packages to effortlessly orchestrate geometry optimizations, frequency calculations, velocity initialization, dynamics propagation, and final signal processing. The paper is organized as follows. “Methods” provides a theoretical summary of the QCT method and details the implementation and underlying architecture of the suite. In “Results and Discussion” we showcase the software's capabilities and provide a hands on guide through a series of model systems: water (H_2_O), formaldehyde (H_2_CO), pyrazine, the acetic acid monohydrate dimer (AcOH ·H_2_O), and the challenging xenon hexafluoride (XeF_6_). Finally, “Conclusions” gathers our concluding remarks and outlines future developments.

## Methods

The detailed derivation of the method is reported elsewhere.^19,20,23–27^ In short, the quasi-classical trajectory (QCT) approach consists in computing the power spectrum of the normal mode displacement *q*(*t*), the normal mode momentum *p*(*t*) or the Cartesian velocity **v**(*t*), along a classical trajectory initialized at quantized energy. To ensure that the trajectory runs on the quantized phase-space torus, the initial conditions are chosen to match those of a set of uncoupled harmonic oscillators at equilibrium. For this, the total energy is decomposed into kinetic and potential contributions

1 $$E\left(p_{0},q_{eq}\right)=\sum_{i}\hbar \omega_{i}\left(n_{i}+\frac{1}{2}\right)=\sum_{i}\frac{p_{0,i}^{2}}{2}+V\left(q_{eq}\right)$$

where **p**_0_ are the initial momenta, **q**_eq_ are the equilibrium positions, ω_*i*_ is the *i*-th harmonic frequency and *n*_*i*_ is the vibrational quantum number. Since in the classical framework *V*(**r**_eq_) = 0, the quantization condition is met by choosing

2 $$\{\begin{array}{l} p_{0}=\sqrt{\hbar \omega \left(2n+1\right)}\\ q_{0}=q_{eq} \end{array}$$

where ω is the vector containing the harmonic frequencies. The computation of the harmonic frequencies ω and of the normal modes **q** is performed after a geometry optimization, using the standard ab initio software methods.

The trajectory is evolved using Born–Oppenheimer molecular dynamics (BOMD), and the QCT power spectrum is computed as the square module of the Fourier transform of the desired time-dependent quantity *f*(*t*)^25–27^

3 $$I_{TA}\left(\omega \right)=\frac{1}{2T}{\left|\int_{0}^{T}dtf\left(t\right)e^{i\omega t}\right|}^{2}$$

where *f*(*t*) can either be the *i*-th normal mode *q*_*i*_, the *i*-th momentum *p*_*i*_, or the Cartesian velocity vector **v**. Equation 3 is the time-averaged version of the more common Fourier transform of the autocorrelation function

4 $$I\left(\omega \right)=\int_{-\infty }^{+\infty }dte^{i\omega t}\lim_{T\to 0}\frac{1}{T}\int_{0}^{T}dt_{0}\left\langle f\left(t_{0}+t\right)f\left(t_{0}\right)\right\rangle$$

Using eq 3, one can obtain the anharmonic spectrum of a molecule using a short, tailored trajectory. The results greatly depend on both the trajectory and the potential energy surface quality, so QCT is a useful instrument to appraise it. However, by construction, it cannot simulate quantum effects, such as zero-point energy, tunneling splitting, and quantum combination bands.^15,26^

The full workflow of a QCT simulation is represented in Figure 1, and it relies on the electronic structure software to optimize the geometry, compute the harmonic frequencies, and perform the dynamics. The Dragonball suite is designed to handle the math and the I/O requirements of such a workflow.

**Figure 1:**
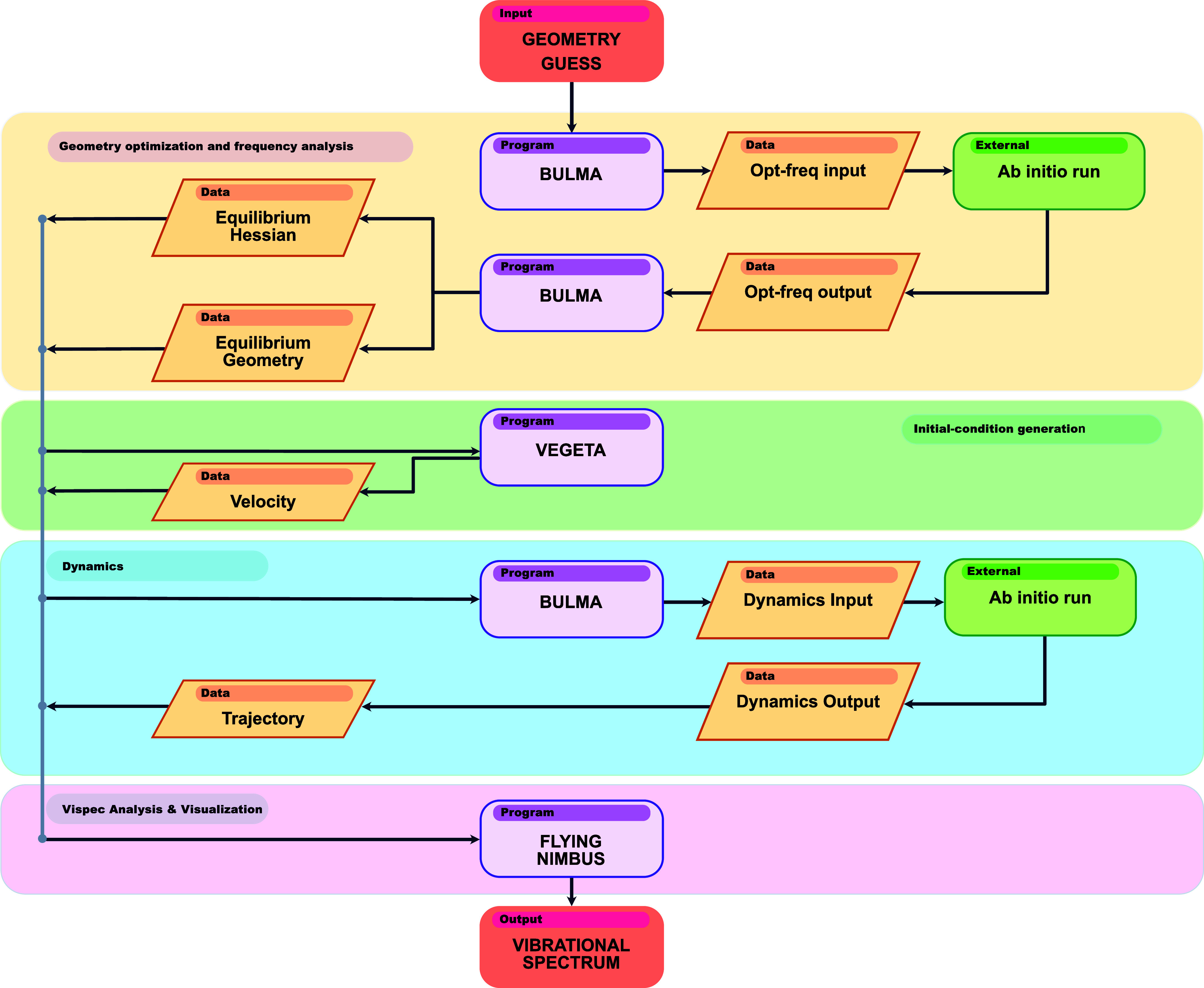
Dragonball flowchart.

Vegeta (Velocity Generator for Time-Averaged Quasiclassical Spectra) takes the equilibrium Hessian matrix (from the frequency calculation) and optimized geometry (from the optimization calculation) to compute the initial velocities following eq 2. Vegeta reads the equilibrium geometry and Hessian from Bulma’s standard output, and outputs the initial velocities for Bulma to read.

Bulma (Batch Utilities for Log parsing, Hessian Matrix and Automation) generates the optimization input from the guess geometry, the frequency input from the optimized geometry, and the BOMD input from the equilibrium geometry and Vegeta’s output velocity. To do so, Bulma just requires a handful of keywords to generate the correct input file for the quantum chemistry code. This is particularly important for providing the quantized initial conditions for the dynamics run. Moreover, it extracts and prepares the equilibrium Hessian for Vegeta, and it collects the trajectory in the $Flying\nu_{i}mbus$ input format, from the various ab initio outputs. Again, this step is crucial since there is no standard output format for this data, and Bulma is required to parse the different formats to provide a single input format for Vegeta and $Flying\nu_{i}mbus$ .

$Flying\nu_{i}mbus$ computes the desired power spectra using the equilibrium geometry, the equilibrium Hessian, and the trajectory, as organized by Bulma. In its GUI form, $Flying\nu_{i}mbus$ also allows to user to plot and analyze the spectra in a pop-up window, where the user can extract the frequency and the fwhm with a handy snap-on-point tool.

In Figure 2, present some screenshots of each application GUI. At the moment of writing, Dragonball is compatible with Gaussian, Orca and QChem,^28–30^ but it can be easily expanded to other quantum chemistry software. Indeed, the user can add to Bulma the functions needed to write the inputs and read the outputs of the desired software, following the software syntax. The complete implementation is described in the Supporting Information.

**Figure 2:**
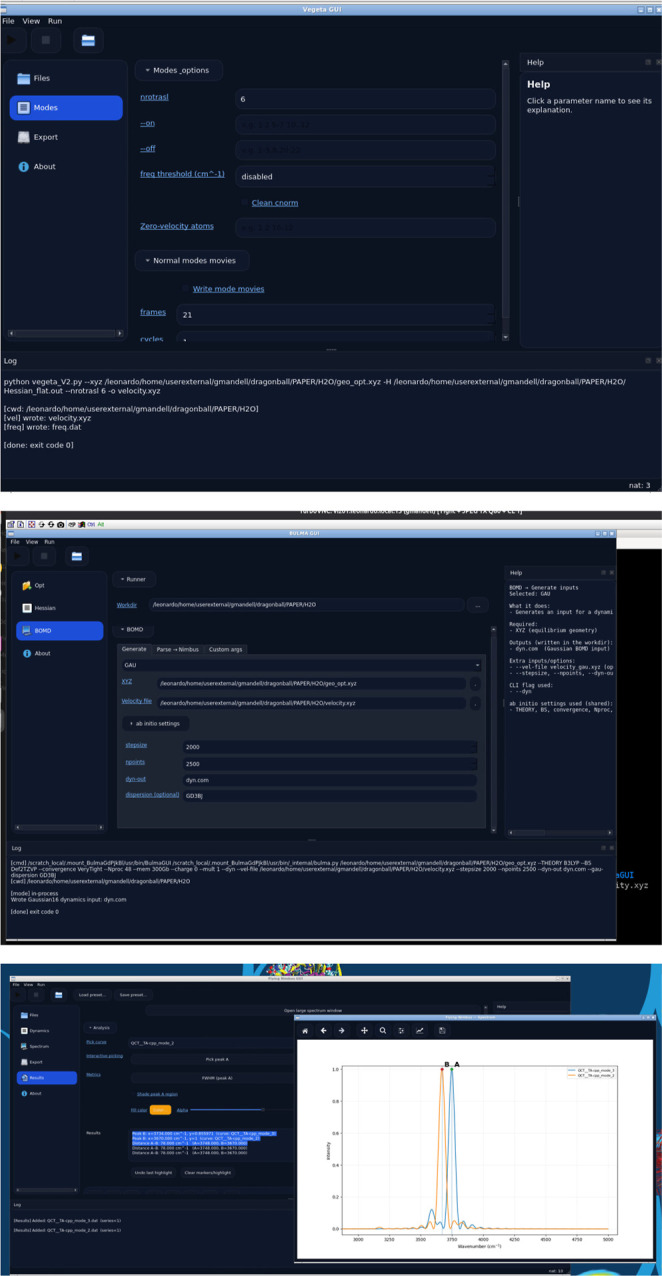
Some screenshots of Dragonball GUIs. From top to bottom: Vegeta’s normal mode selection screen; Bulma’s Born–Oppenheimer dynamics input page; $Flying\nu_{i}mbus$ ’s peak maxima acquisition feature.

## Results and Discussion

The advantages of QCT are well described in the literature.^19,20,25,26,31^ For this reason, we present systems that are of interest to the community, either for learning how to use the Dragonball suite, or for using QCT results in their work. The setup of each simulation is summarized in Table 1.

**Table 1:** Summary of the Levels of Theory Employed in Each Simulation; B3LYP-D4 def2-TZVP is the Default Level of Theory in Dragonball

system	Gau	QChem	Orca
H_2_O	RB3LYP-GD3BJ def2-TZVP	B3LYP-D4 def2-QZVPD	B3LYP-D4 def2-TZVP
H_2_CO	RB3LYP-GD3BJ def2-QZVP	B3LYP-D4 def2-TZVP	B3LYP-D4 def2-TZVP
pyrazine	RB3LYP-GB3DJ aug-cc-pVDZ	B3LYP-D4 def2-TZVP	B3LYP-D4 def2-TZVP
AcOH ·H_2_O	MP2 aug-cc-pVDZ	B3LYP-D4 def2-TZVP	B3LYP-D4 def2-TZVP
XeF_6_	M062X def2-TZVP	B3LYP-D4 def2-TZVP	B3LYP-D4 def2-TZVP

### Water

We simulated the QCT spectra of H_2_O to provide the users a hands-on tutorial on the basic Dragonball workflow. Water, as a small system of great interest, is perfect for this purpose. In Table 2, we report the QCT frequency obtained with three different ab initio codes, compared with the experimental IR frequencies.^32^ In Figure 3, we compare the normal mode results with the full Cartesian spectrum and the experimental frequencies.

**Table 2:** Experimental Frequencies of Gas-phase H_2_O,^32^ Compared with the Harmonic and QCT Frequencies Obtained Using the Dragonball Suite; the Harmonic Frequencies are Taken from Vegeta freq.dat Output; the QCT Frequencies are Taken from the Maxima of the Normal Modes VDOS

		Gaussian		QChem		Orca	
mode	expt. (cm^–1^)	harm (cm^–1^)	QCT (cm^–1^)	harm (cm^–1^)	QCT (cm^–1^)	harm (cm^–1^)	QCT (cm^–1^)
bend	1595	1617	1586	1630	1544	1623	1585
Sym str	3657	3786	3670	3813	3575	3782	3663
Anti str	3756	3891	3748	3913	3645	3883	3735
MAE		95	10	116	81	93	12

**Figure 3:**
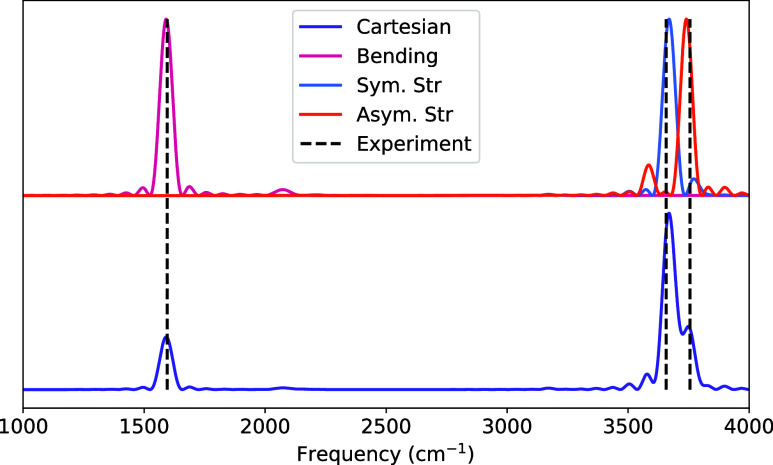
H_2_O QCT spectra obtained from a standard Dragonball run using Orca: at the bottom, the full Cartesian VDOS; on top, the normal modes spectra; the dashed lines correspond to the experimental frequencies.^32^

### Formaldehyde

Formaldehyde is another favorite model system, mainly for the availability of analytical potential energy surfaces (PES).^33,34^ This allowed the study of the vibrational density of states using semiclassical dynamics and adiabatic switching quantization.^19,35^ With H_2_CO, we show that by running a single ab initio trajectory one can still recover the classical vibrational density of states, without the need for an analytical PES and with an acceptable loss in accuracy. In Table 3, we compare our simulation results with the experimental ones,^32^ and the very recent NEO-QCT results,^36^ while in Figure 4 we report the spectra.

**Table 3:** Experimental Frequencies of Gas-phase H_2_CO,^32^ Compared with the Harmonic and QCT Frequencies Obtained Using the Dragonball Suite, Alongside the Recent NEO-QCT Results from Aieta et al.^36^ the Harmonic Frequencies are Taken from Vegeta freq.dat Output; the QCT Frequencies are Taken from the Maxima of the Normal Modes VDOS

		Gaussian		QChem		Orca		Aieta et al.^36^
mode	expt. (cm^–1^)	harm (cm^–1^)	QCT (cm^–1^)	harm (cm^–1^)	QCT (cm^–1^)	harm (cm^–1^)	QCT (cm^–1^)	NEO-QCT (cm^–1^)
CH_2_ wag	1167	1203	1187	1200	1151	1197	1179	1115
CH_2_ rock	1249	1269	1269	1267	1227	1262	1239	1303
CH_2_ scis	1500	1533	1529	1535	1482	1528	1447	1534
CO str	1746	1817	1805	1820	1747	1815	1801	1827
CH_2_ s-str	2783	2881	2763	2883	2677	2882	2738	2894^a^
CH_2_ a-str	2843	2938	2819	2938	2743	2938	2849	2703^a^
MAE	-	59	29	59	44	56	30	79

a Reported as in the paper, even if a better match with the experiment could be obtained by inverting the assignment.

**Figure 4:**
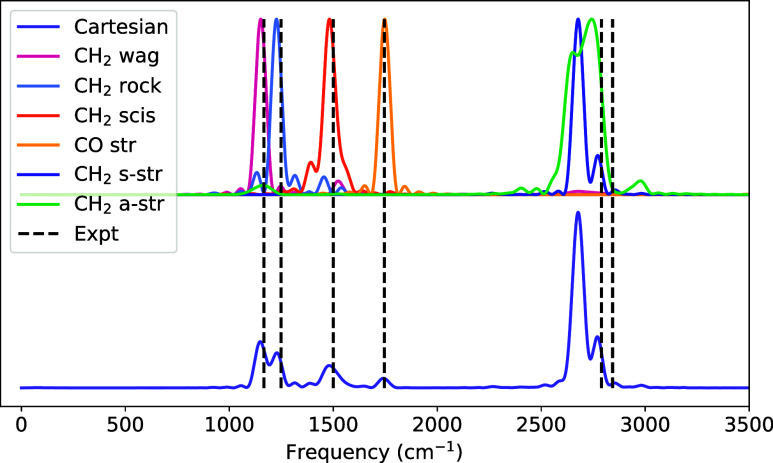
H_2_CO QCT spectra obtained from a standard Dragonball run using QChem: at the bottom, the full Cartesian VDOS; on top, the normal modes spectra; the dashed lines correspond to the experimental frequencies.^32^

For these spectra, one can notice that the system exhibits strong coupling between the two CH_2_ stretching modes (modes 5 and 6) due to their similar energies. When analyzing full-dimensional classical trajectories, this anharmonic mixing often leads to highly congested global power spectra. For this reason, extracting frequencies from the total dipole autocorrelation function or full Cartesian VDOS alone makes peak assignment highly susceptible to misinterpretations. For example, two coupled modes may appear in the spectrum as a doublet, making it impossible to complete the assignment with absolute certainty.^19,36^

The Dragonball suite natively circumvents this issue, because $Flying\nu_{i}mbus$ explicitly computes the power spectrum of the projected normal mode displacements *q*_*i*_(*t*) or momenta *p*_*i*_(*t*) (eq 2), the spectral features are more clearly mapped to specific atomic motions. As a result, the normal mode VDOS should intrinsically prevent misassignments and correctly recover the symmetric stretch at a lower frequency than the asymmetric stretch, safely preserving the correct chemical insight. The exact peak resolution, however, still depends on the potential energy surface and on the trajectory, as one can notice by looking at the spectra obtained using Gaussian’s RB3LYP-GD3BJ def2-QZVP, available on Zenodo.

### Pyrazine

The 24D Pyrazine has become one of the most studied molecular systems in nonadiabatic dynamics,^37–40^ thanks to the accurate vibronic-coupling model Hamiltonian by Raab et al.^41^ Indeed, accounting for the vibrational spectator modes is fundamental to recover the full behavior of the system, in both closed and open quantum systems.^6–9^ For the simulation’s sake, the full VDOS is needed, because the active bath experiences all the spectator modes and not only the optically active ones. However, the knowledge of which modes are IR active is necessary to assign the spectrum of the highly symmetric (*D*_2*h*_) pyrazine. This is where the normal mode decomposition of the full VDOS comes to help, since it can be easily compared with the double harmonic intensities. For this reason, we computed all the normal mode components of the VDOS, but used only the IR-active ones for the comparison with the experimental spectrum.^42^ The comparison of the simulated spectra with the experimental frequencies is shown in Figure 5. Table 4 summarizes the comparison for all the ab initio codes used in the simulations.

**Figure 5:**
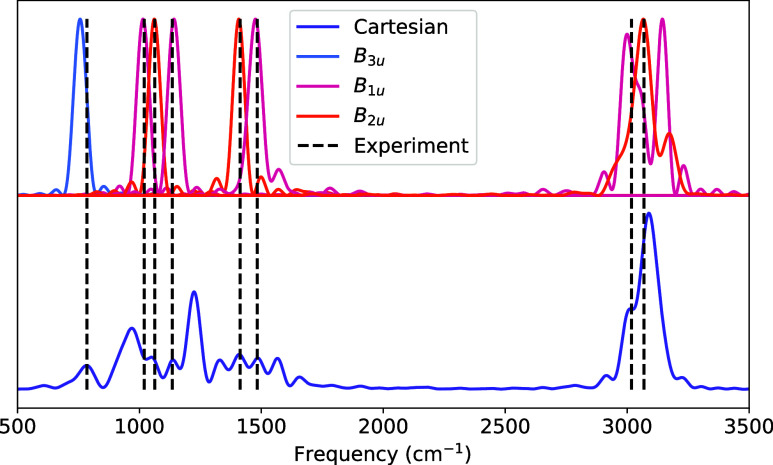
Pyrazine QCT spectra obtained from a standard Dragonball run using Gaussian: at the bottom, the full Cartesian VDOS; on top, the IR-active normal modes spectra; the dashed lines correspond to the experimental frequencies.^42^

**Table 4:** Experimental Frequencies of Pyrazine,^42^ Compared with the Harmonic and QCT Frequencies Obtained Using the Dragonball Suite; the Harmonic Frequencies are Taken from Vegeta freq.dat Output; the QCT Frequencies are Taken from the Maxima of the Normal Modes VDOS

		Gaussian		QChem		Orca	
mode	expt. (cm^–1^)	harm (cm^–1^)	QCT (cm^–1^)	harm (cm^–1^)	QCT (cm^–1^)	harm (cm^–1^)	QCT (cm^–1^)
*B*_3*u*_	785	777	757	781	747	782	753
*B*_1*u*_	1020	1034	1015	1037	982	1037	1015
*B*_2*u*_	1063	1088	1061	1093	1034	1092	1061
*B*_1*u*_	1135	1162	1142	1169	1103	1166	1133
*B*_2*u*_	1413	1435	1408	1447	1353	1445	1399
*B*_1*u*_	1483	1505	1476	1520	1424	1517	1483
*B*_1*u*_	3018	3161	3144	3156	2980	3153	3033
*B*_2*u*_	3069	3176	3066	3170	2974	3166	3036
MAE		46	23	49	49	47	13

### Acetic Acid Monohydrate

The vibrational spectroscopy of organic monohydrates has grown in importance, also thanks to the introduction of jet-expansion, state-of-the-art experiments.^43–45^ This technique was employed to collect some of the experimental data in the HyDRA blind challenge, where QCT performed quite well among the “fully anharmonic” methods.^20^ In this kind of system, it may be useful to separate the spectrum using the atoms instead of the normal modes, since coupling may affect the quality of the simulated signals. This can be easily done with $Flying\nu_{i}mbus$ , using the so-called “atom-wise decomposition”. The implementation of this decomposition is discussed in the Supporting Innformation. As a test case, we studied the AcOH·H_2_O dimer, comparing it with the matrix-isolation experiment.^46^ By decomposing the Cartesian spectrum, we can separate the water and AcOH contributions to the spectrum. This could help gain actual molecular insights from a full-dimensional simulation. In Table 5, we compare the experimental frequencies with those extracted from our simulations. The spectra are then shown in Figure 6. From the graphical comparison, one can notice that the spectrum of each monomer still shows some of the other monomer contributions, due to coupling. This is expected since the underlying simulations remain full-dimensional. However, one can now disentangle signals from heavily coupled normal modes. One disadvantage is that some signals may not be intense enough to be appreciated in the Cartesian picture, as AcOH’s CO and OH str in Figure 6. In this case, a normal mode decomposition can be performed on the same trajectory, obtaining the spectra collected in the additional data at almost no additional computational cost.

**Table 5:** Experimental Frequencies of Ar-Matrix AcOH·H_2_O,^46^ Compared with the Harmonic and QCT Frequencies Obtained Using the Dragonball Suite; the Harmonic Frequencies are Taken from Vegeta freq.dat Output; the QCT Frequencies are Taken from the Maxima of the Normal Modes VDOS; in Cursive, the Assignments That Do Not Agree with the Experimentalists^46^ ^,^ ^a^

		Gaussian		QChem		Orca	
mode	expt. (cm^–1^)	harm (cm^–1^)	QCT (cm^–1^)	harm (cm^–1^)	QCT (cm^–1^)	harm (cm^–1^)	QCT (cm^–1^)
COH oop bnd	858	908	827	915	793	903	820
CH_3_ wag	1001	1014	985	1021	987	1022	999
CH_3_ oop bnd	1052	1058	1040	1072	1015	1072	1046
C–O str	1269	1291	1244	1297	1217	1291	1248
COH bnd	1431	1445	1390	1454	1362	1446	1414
C=Ostr	1732	1759	1681	1769	1691	1765	1745
OH str	3208	3407	3399	3328	3004	3352	3357
H_2_O bnd	1545–1580	1621	1581	1626	1532	1625	1590
OH str	3534	3631	3505	3584	3379	3571	3741
MAE		52	44	45	76	42	51

a The experimental water bending frequency was taken as 1580 c ^–1^ for the computation of the MAE.

**Figure 6:**
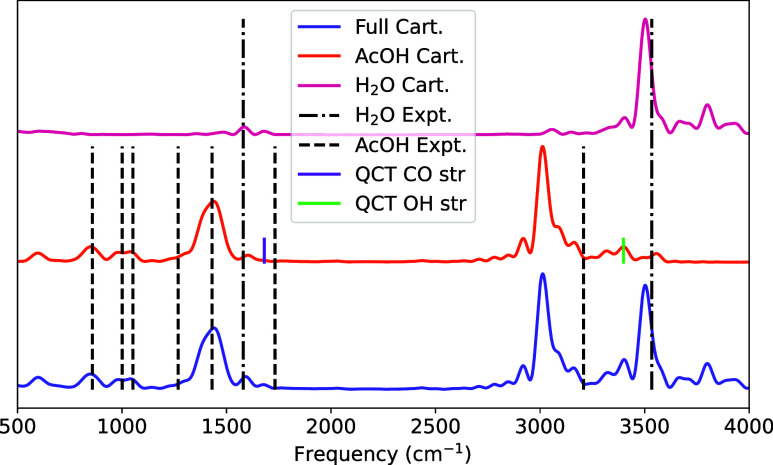
AcOH·H_2_O QCT spectra obtained from a standard Dragonball run using Gaussian: at the bottom, the full Cartesian VDOS; in the middle, the Cartesian spectrum of AcOH in AcOH ·H_2_O, with highlighted the frequencies of the CO and OH stretching as obtained from the normal modes QCT spectra; on top, the Cartesian spectrum of H_2_O in AcOH ·H_2_O; the dashed lines correspond to the AcOH in AcOH ·H_2_O experimental frequencies, the dot-dashed line to those of H_2_O in AcOH·H_2_O.^46^

The atom-wise decomposition could be of great help in studying the effects of microsolvation. In the future, this decomposition could be used to separate the spectra of the solute and of the solvent, in a manner not dissimilar from the “Solute Correlated” spectra.^47^ Finally, a QM-MM or ONIOM approach could be used to simulate the spectra in matrix isolation, as those of AcOH·H_2_O, to properly account for the matrix effects. In this case, the atom-wise decomposition is necessary to remove the unwanted signal from the matrix itself.

### Xenon Exafluoride

One of the main advantages of in silico simulation is that it can easily compute the spectra of systems that can be difficult to isolate experimentally. To show this, we simulated the spectra of XeF_6_, which ideally could be obtained with no more pain than those of the previous systems. For Xe, all of the chosen ab initio codes automatically select an effective core potential (ECP) basis set when using a Karlsruhe basis.^48^ In Table 6, we summarize the frequency comparison with the experiment.^49^ The simulated frequencies do not match well the experiment, due to several factors. First of all, the experimental data are not recent, and the spectra were not obtained in the gas phase but in a cryogenic Ar matrix, which can play an important role in the outcome.^50,51^ Then, the selected level of theory and basis set may not be the best possible for this molecule. In addition to this, XeF_6_ vibrational spectrum falls in a range of frequencies (0–1000 c ^–1^) that stretches the sampling power of the short ab initio trajectory used for QCT.

**Table 6:** Experimental Frequencies of Ar-Matrix XeF_6_,^49^ Compared with the Harmonic and QCT Frequencies Obtained Using the Dragonball Suite; the Harmonic Frequencies are Taken from Vegeta freq.dat Output; the QCT Frequencies are Taken from the Maxima of the Normal Modes VDOS

assign. From ref 49				Gaussian (deformed)		QChem (octahedral)		Orca (octahedral)	
Oct	Obl	Prol	expt. (cm^–1^)	harm (cm^–1^)	QCT (cm^–1^)	harm (cm^–1^)	QCT (cm^–1^)	harm (cm^–1^)	QCT (cm^–1^)
				217	201	120	165	128	151
				269	262	123	145	134	163
				274	262	138	127	144	142
*f*_1*u*_			252	279	263	149	152	153	131
	*e*_*u*_		302	382	374	201	168	206	222
	*a*_2*u*_		326	384	380	207	263	211	177
		*a*_2*u*_	352	388	378	214	210	214	169
		*e*_*u*_	365	535	531	492	478	498	497
	*e*_*u*_		384	536	529	499	478	506	494
	*e*_*u*_		506	592	570	560	547	563	572
electronic	557	685	675	567	539	573	557		
*f*_1*u*_			624	687	675	572	551	576	572
	*a*_2*u*_		630	710	698	573	559	586	562

We point out that we wanted to be consistent, so we did not overcome these limitations with ad hoc empirical scaling of the vibrational spectra. In static calculations, it is common practice to apply standard scaling factors to harmonic frequencies to simultaneously correct for both electronic structure errors and the lack of mechanical anharmonicity. However, because QCT inherently captures full-dimensional anharmonicity and mode-coupling on-the-fly, applying harmonic scaling factors to QCT spectra risks double-counting anharmonic effects and masks the true quality of the underlying ab initio potential energy surface. Despite this, some recent NEO dynamics-based studies have resorted to empirical scaling to force visual alignment with the experiment.^36^ The automated workflow of Dragonball strictly outputs the raw VDOS, without including any scaling factor to the frequencies, since QCT has proven to be quite accurate.^20^ Moreover, only by reporting the unscaled vibrational results, one can properly attest the limitations of the method and of the electronic structure theory, providing an unbiased foundation for a systematic improvement of ab initio methods and fitted PES. After a cursory analysis of the frontier orbital energies (Table 7) we question the experimental assignment of the electronic transition at 557 c ^–1^. This suggests that the full assignment of this vibrational spectrum would require care that is beyond the scope of this paper.

**Table 7:** Comparison of Frontier Orbital Energies and TD-DFT Excitation Gaps for XeF_6_ Computed with Q-Chem, ORCA and Gaussian16

code	*E* HOMO (Eh)	*E* LUMO (Eh)	Δ*E* (Eh)
QChem	–0.3799	–0.1788	0.2011
Orca	–0.3760	–0.1834	0.1926
G16	–0.4379	–0.1095	0.3284
	10 roots	0.1174	
QChem TD-DFT	20 roots	0.1174	
	50 roots	0.1246	

The simulation results may thus be improved either by decomposing the spectrum with other vibrational coordinates,^52^ or by pushing the spectral analysis beyond the capabilities of the simple Fourier transform. These techniques will be addressed and implemented in a future release we are working on. While these applications would surely improve the quality and readability of the results, more accurate results could be obtained by including the matrix into the simulation, and by properly accounting for the many conformers of XeF_6_.

## Conclusions

In this paper, we presented Dragonball, a suite for the simulation of ab initio vibrational density of states (VDOS) using the quasi-classical trajectory (QCT) method. In its ab initio instance, QCT uses a single classical trajectory initialized at quantized energy to obtain the anharmonic vibrational frequencies of the molecule. As the interest in methods that account for vibrational anharmonicity grows,^20^ and with an increase in the computational power available for quantum chemistry, QCT is becoming more and more a viable method in vibrational spectroscopy. Some tools are already available, such as the SEMISOFT web platform created by Ceotto and co-workers.^22^ However, the SEMISOFT Web site does not provide any support in actually running the quasi-classical trajectory, which we deem the trickiest task of the work. Furthermore, documentation and assistance are also provided with additional capabilities implemented in the graphical and mathematical postprocessing of the dynamics.

Dragonball provides a ready-to-use, user-friendly Python toolbox that guides the user from guess geometry to vibrational spectrum. The three codes, Vegeta, Bulma and $Flying\nu_{i}mbus$ are available both as Python scripts, for a quick CLI workflow, and as standalone executables, with an easy GUI. The suite encompasses all the steps of the QCT workflows and works locally, so that no data movement is required, as with other platforms. Finally, the suite is available on GitHub under the PolyForm-Noncommercial license, for anyone to use for personal and research purposes. The documentation is available in the Supporting Information, in the repository, and on the Web site (https://dragonball-vispec.readthedocs.io/en/latest/index.html).

The QCT framework and Dragonball code structure allow for easy adoption, but also for easy customization and improvement. On the simulation side, we are currently working on including the matrix and solvent effect through ONIOM simulations. On the analysis side, we are investigating the adoption of new vibrational coordinates beyond the simple normal modes and the implementation of different and more advanced signal-analysis algorithms. Furthermore, a proprietary classical dynamics integrator will be included to enable the interface with quantum software with more complex ab initio methods (such as DLPNO–CC and MCSCF methods)^53,54^ and use them to compute power spectra.

In parallel, we are building another GUI application to visualize and connect the trajectory and the spectra (Shenron), together with a structured database optimized for big data handling and capable of collecting trajectories and MD information. Structured and personalized queries will be possible and will facilitate the integration with experimental data. The Dragonball and Shenron infrastructure will allow the computation of spectroscopic and statistical parameters from on-the-fly simulations, and provide the vibrational spectroscopy community with the data from molecular dynamics simulations. A validation system will allow the users to include their Dragonball standard outputs in the database.

## Supplementary Material



## Data Availability

The data that support the findings of this study are available in the following repository. The code is available here. The full documentation is available here.

## Notes

The Supporting Information includes the technical implementation of the code and a summary of the most common commands. The raw data created with Dragonball for this paper is available here (PDF)
