# Supplementary material for: DragonBall: A Suite for Vibrational Spectroscopy with Classical Trajectories
Source: J Phys Chem A. 2026 Jul 20;130(30):5962–71. doi: 10.1021/acs.jpca.6c04232 (PMC13430688; doi:10.1021/acs.jpca.6c04232)
Supplement: Supplementary file 1 [file jp6c04232_si_001.pdf]

Supporting Information: DragonBall: a Suite for Vibrational Spectroscopy With Classical Trajectories

Giacomo Mandelli,*,† Antonino Famulari,† Carlo Cavallotti,† Sophya Garashchuk,‡ and Giacomo Botti*,‡

†*Politecnico di Milano, Department of Chemistry, Materials, and Chemical Engineering "Giulio Natta", Piazza Leonardo da Vinci 32, 20133 Milan, Italy*

‡*University of South Carolina, Department of Chemistry and Biochemistry, 631 Sumter St, Columbia, SC 29208, United States of America*

E-mail: giacomo.mandelli@polimi.it; GBOTTI@mailbox.sc.edu

Availability and License

Dragonball is available under PolyForm-Noncommercial license. The *full documentation* of the Dragonball suite is available here. The *source code* is available here. The *Linux Appimages* are available here. The *Windows executables* are available here.

VEGETA

VEGETA stands for Velocity Generator for Time Average Quasiclassical Spectra.

The program generates initial Cartesian velocities (in atomic units) at the equilibrium geometry, starting from a "flat" Hessian matrix (lower-triangular, one number per line, with two blank spaces at the beginning) employed in molecular dynamics for the computation of quasiclassical spectra.

Implementation

In the first step, given the Cartesian Hessian $\mathbb{H} \in \mathbb{R}^{3N \times 3N}$ and Cartesian masses $\{m_i\}_{i=1}^{3N}$, where N is the number of atoms, the mass-weighted Hessian is computed:

$$\widetilde{H}_{ij} = \frac{H_{ij}}{\sqrt{m_i m_j}}. \tag{1}$$

The Hessian is then diagonalized using a Python-implemented version of the EISPACK diagonalizer.

$$\widetilde{\mathbb{H}}\,\mathbf{c}_k = \lambda_k \mathbf{c}_k, \tag{2}$$

with eigenvalues λ_k and eigenvectors $\mathbf{c}_k$ (columns of $\mathbb{C}$, the Cnorm matrix).

Vibrational eigenvalues are taken as the last N_{vib} eigenvalues (N_{vib} is the number of vibrational eigenvectors):

$$\{\lambda_v\} = \lambda_{3N-N_{\text{vib}}+1}, \ldots, \lambda_{3N}. \tag{3}$$

The script reorders eigenvectors so that vibrational modes are first. This layout is assumed throughout all the later steps.

The vibrational frequencies ω_k are then obtained from the eigenvalues:

$$\omega_k = \sqrt{|\lambda_k|} \tag{4}$$

The quantized initial conditions are then computed. For each vibrational mode, the k-th normal-mode momentum p_k is set to

$$p_k = \begin{cases} \sqrt{\omega_k}\,\sqrt{2n_k + 1} & \text{mode active} \\ 0 & \text{mode switched off} \end{cases} \tag{5}$$

where n_k is the k-th vibrational quantum number. Then, given the Cnorm $\mathbb{C}$ matrix, the mass-weighted Cartesian momenta $\mathbf{p}_c$ are defined:

$$\mathbf{p}_c = \mathbb{C}\,\mathbf{p}. \tag{6}$$

And the Cartesian velocities v_k are computed (componentwise, with Cartesian masses m_k):

$$v_k = \frac{(p_c)_k}{\sqrt{m_k}}, \qquad k = 1, \ldots, 3N. \tag{7}$$

The total kinetic energy, useful later for rescaling, is computed as:

$$T = \frac{1}{2}\sum_k m_k v_k^2. \tag{8}$$

At this point, the program can remove the overall rotation using a principal-axes proce-

dure. From the input equilibrium geometry, the inertia tensor is built:

$$\mathbb{I} = \sum_{i}^{N} m_i \begin{pmatrix} y_i^2 + z_i^2 & -x_i y_i & -x_i z_i \\ -x_i y_i & z_i^2 + x_i^2 & -y_i z_i \\ -x_i z_i & -y_i z_i & x_i^2 + y_i^2 \end{pmatrix}. \tag{9}$$

where N is the number of atoms. Then upon diagonalization, the eigenvalues (I_x, I_y, I_z), corresponding to the principal moments of inertia and the eigenvectors (composing a 3×3 rotation matrix $\mathbb{N}$), *i.e.* the principal axes, are obtained:

$$\mathbb{I} = \mathbb{N}\,\mathrm{diag}(I_x, I_y, I_z)\,\mathbb{N}^\mathsf{T} \tag{10}$$

In this way, it is possible to rotate the equilibrium coordinates $\mathbf{r}$ and the Cartesian velocities $\mathbf{v}$ into the principal-axis frame:

$$\mathbf{r}'_a = \mathbb{N}^\mathsf{T}\mathbf{r}_a, \qquad \mathbf{v}'_a = \mathbb{N}^\mathsf{T}\mathbf{v}_a. \tag{11}$$

The rigid body rotational velocity for each atom a is then computed $\mathbf{v}'_{\mathrm{rot},a} = \boldsymbol{\Omega} \times \mathbf{r}'_a$, where $\boldsymbol{\Omega}$ is the angular velocity vector, and the rotation-removed velocities are obtained:

$$\mathbf{v}'_a \leftarrow \mathbf{v}'_a - (\boldsymbol{\Omega} \times \mathbf{r}'_a). \tag{12}$$

At this point, we rotate back to the original frame:

$$\mathbf{v}_a = \mathbb{N}\mathbf{v}'_a, \qquad \mathbf{r}_a = \mathbb{N}\mathbf{r}'_a. \tag{13}$$

and we restore the kinetic energy to the target T_0 by rescaling:

$$\alpha = \sqrt{\frac{T_0}{T'}}, \qquad \mathbf{v} \leftarrow \alpha\,\mathbf{v}. \tag{14}$$

Cnorm Cleaning Procedure

In certain cases, we can force the cleaning of the Cnorm matrix ($\mathbb{C}$) to remove residual mixing of vibrations with roto-translational. The implemented steps take care of this when `--clean-cnorm 1` is set. The first step of the procedure is to split the eigenvector matrix ($\mathbb{C}$) into vibrational $\mathbb{C}_{\nu 0}$ and roto-translational $\mathbb{C}_{rt0}$ blocks. The roto-translational block is then orthonormalized using Gram-Schmidt, thus obtaining the orthonormal basis for the roto-translational subspace $\mathbb{Q}_{\mathrm{rt}} \in \mathbb{R}^{3N\times N_{\mathrm{rt}}}$:

$$\mathbb{Q}_{\mathrm{rt}} = \mathrm{GS}(\mathbb{C}_{\mathrm{rt}0}). \tag{15}$$

Then the candidate vibrational vectors $\mathbb{C}_{\nu 0}$ are projected ortogonal to the roto-translational:

$$\mathbb{V} = \mathbb{C}_{\nu 0} - \mathbb{Q}_{\mathrm{rt}}(\mathbb{Q}_{\mathrm{rt}}^{\mathsf{T}}\mathbb{C}_{\nu 0}). \tag{16}$$

And then orthonormalized to get a vibrational basis:

$$\mathbb{Q}_{\nu} = \mathrm{GS}(\mathbb{V}), \qquad \mathbb{Q}_{\nu}^{\mathsf{T}}\mathbb{Q}_{\nu} = \mathbb{I}_{N_{\nu}}, \quad \mathbb{Q}_{\mathrm{rt}}^{\mathsf{T}}\mathbb{Q}_{\nu} = \mathbf{0}. \tag{17}$$

Now, the mass-weighted Hessian is projected into the vibrational subspace:

$$\mathbb{H}_{\nu} = \mathbb{Q}_{\nu}^{\mathsf{T}}\tilde{\mathbb{H}}\,\mathbb{Q}_{\nu}. \tag{18}$$

and finally diagonalized:

$$\mathbb{H}_{\nu}\,\mathbb{U} = \mathbb{U}\,\mathbf{\Lambda}_{\nu}. \tag{19}$$

and then, going back, the cleaned Cartesian space eigenvectors matrix $\mathbb{C}_{\nu}^{cl}$ is obtained:

$$\mathbb{C}_{\nu}^{cl} = \mathbb{Q}_{\nu}\mathbb{U}. \tag{20}$$

Finally, the cleaned full basis is assembled by adding the rot-translational eigenvectors and force to zero the roto-translational eigenvalues.

$$\mathbb{C}^{cl} = \left[\ \mathbb{C}_{\nu}^{cl}\ \middle|\ \mathbb{Q}_{\mathrm{rt}}\ \right]. \tag{21}$$

From the cleaned eigenvalues, the final vibrational frequencies are obtained:

$$\omega_v^{cl} = \sqrt{|\lambda_v^{cl}|} \tag{22}$$

Flying $\nu_i$mbus

Flying $\nu_i$mbus is a Python program to compute vibrational power spectra from trajectory data. It supports two routes:

- *time-averaged Fourier transform* (TA-FT) power spectra (default), and
- *time-correlation functions* followed by a Fourier transform (enabled with `--no-ta`).

Spectra can be computed either by projecting motion onto mass-weighted normal modes built from the equilibrium Cartesian Hessian (`--coord nm`), or directly in Cartesian space using a velocity autocorrelation power spectrum (`--coord cart`). An optional atom subset selection (`--atoms`) provides partial, atom-resolved spectral contributions taken from the total Cartesian spectrum.

Implementation

The Flying Nimbus program analyzes nuclear trajectories evolved on an electronic potential energy surface (PES) within the Born–Oppenheimer approximation. No dynamics are performed here; the code assumes you already have $\{\mathbf{x}(t), \mathbf{v}(t)\}$ and extracts spectral information from time series. Normal mode analysis is performed first in the same way as

described in VEGETA. Upon definition of the normal mode eigenvectors, the mass-weighted Cartesian coordinates $\tilde{\mathbf{x}}$ and velocities $\tilde{\mathbf{v}}$ are obtained:

$$\tilde{\mathbf{x}}(t) = \mathbf{x}(t) \cdot \sqrt{\mathbf{m}}, \qquad \tilde{\mathbf{v}}(t) = \mathbf{v}(t) \cdot \sqrt{\mathbf{m}}, \tag{23}$$

where $\sqrt{\mathbf{m}}$ repeats $\sqrt{m_i}$ over (x, y, z) components. The normal-mode projections are then defined as:

$$\mathbf{q}(t) = \tilde{\mathbf{x}}^{\mathsf{T}}(t)\,\mathbb{C}, \qquad \mathbf{p}(t) = \tilde{\mathbf{v}}^{\mathsf{T}}(t)\,\mathbb{C}. \tag{24}$$

where $\mathbb{C}$ is the Cnorm matrix (vib-first) as defined in VEGETA chapter. The code uses the first N_{vib} columns of $\mathbf{q}(t)$ and $\mathbf{p}(t)$ for vibrational analysis. It is possible for the user to do an **atom selection**. If the user specifies a subset of atoms, the code builds a Cartesian mask $M \in \{0, 1\}^{3N}$ that keeps only DOFs of selected atoms. The mask is applied **before** projection:

$$\tilde{\mathbf{x}}_{\text{sub}}(t) = \tilde{\mathbf{x}}(t) \cdot M, \qquad \tilde{\mathbf{v}}_{\text{sub}}(t) = \tilde{\mathbf{v}}(t) \cdot M, \tag{25}$$

and then projected with the same eigenvector matrix $\mathbb{C}$ as in the full dimensional case:

$$\mathbf{q}_{\text{sub}}(t) = \tilde{\mathbf{x}}^{\mathsf{T}}_{\text{sub}}(t)\,\mathbb{C}, \qquad \mathbf{p}_{\text{sub}}(t) = \tilde{\mathbf{v}}^{\mathsf{T}}_{\text{sub}}(t)\,\mathbb{C}. \tag{26}$$

Therefore the NM spectra represent the contribution of the selected atoms to the normal-mode motion. On the other hand, in Cartesian mode, atom selection simply restricts which atomic velocities contribute to $C_{vv}(\tau)$ or to the TA power spectrum.

In `--no-ta` mode, (also called here **corr-FT**) the program first builds correlation functions as functions of a lag index then applies a numerical Fourier transform to obtain the spectrum.

Let N_T be the number of available trajectory steps after applying `--nstart`. Let $N_c =$ `ncorr` be the correlation length (maximum lag $N_c - 1$). The code uses a set of time origins (starting

points) called *beads*. The lag index can be defined as:

$$\ell = 0, 1, \ldots, N_c - 1, \qquad \tau_\ell = \ell\,\Delta t, \tag{27}$$

If `--nbeads` is non-positive, it is set automatically to the maximum number of valid origins that still allow a lag up to $N - 1$:

$$n_{\text{beads}} \leftarrow N_T - (N_c - 1). \tag{28}$$

Origins are selected with `--nbeadsstep`:

$$\mathcal{O} = \{0,\, s,\, 2s,\, \ldots\}, \qquad s = \texttt{nbeadsstep}. \tag{29}$$

and the normalization is defined:

$$D = \left(\frac{n_{\text{beads}}}{s}\right) - 1. \tag{30}$$

if $D \leq 0$ it is set to $D = |\mathcal{O}|$.

In **NM mode** the program is called twice:

on $p_m(t)$ (projected velocities): producing $C_{pp}(\tau)$ and `FT-cpp` spectra,

on $q_m(t)$ (projected coordinates): producing $C_{qq}(\tau)$ and `FT-cqq` spectra.

At this point, for a selected mode m and lag ℓ, the products over valid origins are computed:

$$C_m(\ell) = \frac{1}{D} \sum_{t_0 \in \mathcal{O}_{\text{valid}}(\ell)} s_m(t_0 + \ell)\, s_m(t_0), \tag{31}$$

where s_m is either p_m or q_m, and $\mathcal{O}_{\text{valid}}(\ell)$ is the set of t_0 such that

$$\mathcal{O}_{\text{valid}}(\ell) = \{t_0 \in \mathcal{O} \;:\; t_0 + \ell < N_T\}. \tag{32}$$

If $\alpha \neq 0$, the code multiplies each lag by a Gaussian factor

$$C_m(\ell) \leftarrow C_m(\ell)\,\exp\big(-\alpha\,t^2\big), \qquad t = (\ell+1)\Delta t, \tag{33}$$

using $(\ell+1)\Delta t$ (one-step shifted) rather than $\ell\Delta t$.

In Cartesian mode, the code computes a velocity autocorrelation, optionally restricting to selected atoms, for each origin t_0 and lag ℓ, computing per-atom dot products $\mathbf{v}_a(t_0+\ell)\cdot\mathbf{v}_a(t_0)$, then averaging over atoms, then summing over origins and dividing by the normalization D. For each lag:

$$C_{vv}(\ell) = \frac{1}{D}\sum_{t_0\in\mathcal{O}_{\text{valid}}(\ell)}\left[\frac{1}{N_{\text{sel}}}\sum_{a=1}^{N_{\text{sel}}}\mathbf{v}_a(t_0+\ell)\cdot\mathbf{v}_a(t_0)\right]. \tag{34}$$

This yields one scalar correlation function $C_{vv}(\tau)$.

In TA route (default) the code does not compute $C(\tau)$. Instead, it takes the first $N =$ `ncorr` points of the relevant time series (or velocity components) and computes a power spectrum directly.

All transforms are performed on a uniform time grid as defined in Eq. 27, while spectra are written on a linear *wavenumber* grid (cm^{-1}):

$$\tilde{\nu}_k = \tilde{\nu}_0 + k\,\Delta\tilde{\nu}, \qquad k = 0,\ldots,N_f-1, \tag{35}$$

with $\tilde{\nu}_0 =$ `init_wnumb`, $\Delta\tilde{\nu} =$ `spec_res`, and

$$N_f = \left\lfloor \frac{\texttt{wnumb\_span}}{\texttt{spec\_res}} \right\rfloor. \tag{36}$$

The FT is computed as discrete Fourier Transform using Simpson numerical quadratue.

The numerical time integral of the form

$$\mathcal{F}(\omega) = \int_0^T g(t)\, e^{i\omega t}\, dt, \qquad T = (N-1)\Delta t, \tag{37}$$

is approximated by a weighted sum on the discrete grid:

$$\mathcal{F}(\omega_k) \approx \sum_{j=0}^{N-1} w_j\, g(t_j)\, e^{i\omega_k t_j}. \tag{38}$$

The weights w_j correspond to Simpson's 1/3 rule pattern:

$$w_j \propto \{1, 4, 2, 4, 2, \ldots, 4, 1\}, \tag{39}$$

with endpoints equal to 1 and interior points alternating 4 and 2. In the implementation, the factor $\Delta t/3$ is included directly in the returned weights, so w_j already has units of time:

$$w_j = \frac{\Delta t}{3} \times \begin{cases} 1, & j = 0 \text{ or } j = N-1, \\ 4, & j \text{ odd}, \\ 2, & j \text{ even (interior)}. \end{cases} \tag{40}$$

Note that computing the complex exponent matrix for all N_f points at once would require building a potentially large complex matrix $\mathbf{E}$ of shape $(N_f \times N)$. To reduce peak memory usage, the implementation computes the FT via frequency blocks of size `block` (default 256). So, we obtain the complex arrays $\mathbf{F}_{\text{block}}$ and, upon reconstruction, the final $\mathbf{F}(\omega_k)$.

Finally, with the `--no-ta` option the program computes correlations $C(\tau)$ first, then transforms them. The printed intensity uses the magnitude of the real part:

$$I_{\text{FT}}(\omega_k) = \text{Re}[\mathcal{F}(\omega_k)]. \tag{41}$$

Thus FT intensities are in arbitrary units and consistent only within the same normalization.

For the Time-averaged option (TA-FT, default), the program transforms the time series directly and produces a power spectrum:

$$I_{\mathrm{TA}}(\omega_k) = \frac{|\mathcal{F}(\omega_k)|^2}{2\,(N-1)\Delta t}. \tag{42}$$

For Cartesian velocities, the code sums the power over all transformed components before applying the same prefactor.

Detailed Options

BULMA

**Option**	**Default**	**Meaning / Notes**
**Core input/output (always available)**		
`input_file`	required	Positional input path. Its meaning depends on the selected mode:<br>• **Default (no mode flags):** Gaussian output `.out`/`.log` for Hessian extraction.<br>• `--orca-hess`**:** ORCA `.hess` file.<br>• `--qchem-hess`**:** Q-Chem `HESS` file.<br>• **Any generator mode (e.g.** `--opt`**,** `--freq`**,** `--dyn`**, . . .):** equilibrium `.xyz`.<br>• **Parsers (**`--parse-dyn`**,** `--parse-qchem-qmd`**):** typically the corresponding output file (unless overridden by dedicated options).

Option	Default	Meaning / Notes
`-m, --matrix-out`	`Hessian.out`	Output Hessian file in **lower-triangular row format** (written with Fortran `D` exponents). Used in Hessian-extraction modes.
`-v, --vector-out`	`Hessian_flat.out`	Output Hessian flattened to a **1-column vector**, preceded by two blank lines, written with `D` exponents. Used in Hessian-extraction modes.
**Hessian extraction**		
`--orca-hess`	off	Extract Hessian from an ORCA `.hess` file (the positional `input_file` is the `.hess`). Internally rebuilds the full matrix and then writes the lower triangle to outputs.
`--qchem-hess`	off	Extract Hessian from a Q-Chem `HESS` file (the positional `input_file` is `HESS`). Reads `$hessian` . . . `$end` and writes lower triangle to outputs.
**Mutually exclusive *main modes* (choose at most one)**		
`--opt`	off	Generate Gaussian16 geometry optimization input `geom.com` from XYZ (`input_file` is XYZ).
`--freq`	off	Generate Gaussian16 frequency-job input `geom_freq.com` from XYZ (`input_file` is XYZ).
`--orca-opt`	off	Generate ORCA optimization input `geom.inp` from XYZ (`input_file` is XYZ).
`--orca-freq`	off	Generate ORCA opt+freq input `geom_freq.inp` from XYZ (`input_file` is XYZ).
`--dyn`	off	Generate Gaussian16 BOMD input `dyn.com` from equilibrium XYZ (`input_file`) plus velocities read from `--vel-file`.

Option	Default	Meaning / Notes
`--orca-qmd`	off	Generate ORCA QMD inputs `<prefix>_qmd.inp` and `<prefix>_qmd.mdrestart` from equilibrium XYZ (`input_file`) plus velocities from `--orca-vel-file`.
`--qchem--qmd`	off	Generate Q-Chem QMD input `dyn.inp` (or `--qchem-qmd-out`) from equilibrium XYZ (`input_file`) plus velocities from `--qchem-vel-file`.
`--qchem-opt-single`	off	Generate Q-Chem *single-job* OPT input `opt.inp` from XYZ (`input_file`).
`--qchem-freq`	off	Generate Q-Chem *single-job* FREQ input `freq.inp` from XYZ (`input_file`).
`--qchem-opt`	off	Generate Q-Chem *multi-job* optimization input `opt.inp` from XYZ (`input_file`).
`--qchem-opt-freq`	off	Generate Q-Chem *multi-job* opt+freq input `opt-freq.inp` from XYZ (`input_file`).
**Common electronic-structure settings (used by generators)**		
`--BS`	`Def2TZVP`	Basis set (Gaussian/ORCA/Q-Chem).
`--THEORY`	`B3LYP`	Method keyword (Gaussian/ORCA/Q-Chem).
`--convergence`	`VeryTight`	Convergence keyword used in geometry optimization (Gaussian `Opt=(...,<conv>)`; ORCA maps to an OPT keyword).
`--Nproc`	48	Requested number of cores (`%NProcShared` in Gaussian; `%PAL NPROCS` in ORCA).
`--mem`	`300Gb`	Requested memory string. Used by Gaussian as `%mem`. ORCA may convert it to `%maxcore` (MB/core) in some modes.
`--charge`	0	Total charge.

Option	Default	Meaning / Notes
`--mult`	1	Spin multiplicity.
**Geometry extraction (from outputs; skips Hessian extraction)**		
`--extract-geo`	off	Extract the last optimized geometry and exit (no Hessian extraction). Auto-detects: Gaussian last `Standard orientation`, ORCA last `CARTESIAN COORDINATES (ANGSTROEM)` after convergence, Q-Chem last `Standard Nuclear Orientation (Angstroms)`.
`--geo-out`	`geo_opt.xyz`	Output XYZ filename for extracted geometry.
`--xyz-template`	`geo.xyz`	Template XYZ providing atom symbols/order for geometry output (needed particularly for Gaussian extraction, where the standard orientation table lacks symbols).
**Gaussian BOMD input generation (`--dyn`)**		
`--stepsize`	2000	Gaussian BOMD `StepSize` used in `BOMD(...)`.
`--npoints`	2500	Gaussian BOMD `MaxPoints` (number of MD points).
`--vel-file`	`velocity_gau.xyz`	Velocity file used by `--dyn`. Format: XYZ-like header + per-atom `vx vy vz` (last 3 tokens per line are used).
`--dyn-out`	`dyn.com`	Output filename for Gaussian BOMD input.
`--chk`	none	Checkpoint filename for Gaussian BOMD input. If omitted, a default name is used internally.
**ORCA QMD input generation (`--orca-qmd`)**		
`--orca-vel-file`	`velocity_orca.xyz`	Velocity file for ORCA QMD generation (XYZ-like header; last 3 tokens per atom line are used).

Option	Default	Meaning / Notes
`--orca-vel-unit`	`au`	Units of the velocity file for ORCA restart writing: `au` (bohr/au_time) or `angfs` (Å/fs). Restart velocities are written in Å/fs.
`--qmd-timestep`	0.20	ORCA MD timestep in fs for the generated QMD input.
`--qmd-run`	2501	Number of ORCA MD steps in the generated QMD input.
`--qmd-prefix`	none	Prefix for `<prefix>_qmd.inp` and `<prefix>_qmd.mdrestart`. If not set, uses the XYZ stem.

Q-Chem AIMD/QMD input generation (`--qchem-qmd`)

`--qchem-vel-file`	`velocity.xyz`	Velocity file for Q-Chem AIMD generation (XYZ-like header; last 3 tokens per atom line are used). Velocities are written *as provided* (normalized to `E` exponent internally).
`--qchem-qmd-out`	`dyn.inp`	Output filename for Q-Chem AIMD input.
`--qchem-timestep`	8	Q-Chem `Time_step` in atomic units of time.
`--qchem-steps`	2500	Q-Chem `aimd_steps`.
`--qchem-print`	1	Q-Chem `aimd_print` (print frequency).

Gaussian BOMD output parsing (`--parse-dyn`)

`--parse-dyn`	off	Parse Gaussian BOMD dynamics from an output/log and write processed trajectories/energies. Requires `--inizio`, `--fine`, and `--xyz`.
`-i, --inizio`	none	Starting step (inclusive) for parsing. Required with `--parse-dyn`.
`-f, --fine`	none	Ending step (exclusive) for parsing. Required with `--parse-dyn`.

Option	Default	Meaning / Notes
`-N, --Natom`	none	Optional atom count check. If provided, must match the number of atoms in `--xyz`.
`-g, --gout`	none	Gaussian output file to parse. If omitted, uses the positional `input_file`.
`--xyz`	none	Reference equilibrium XYZ (required with `--parse-dyn`); provides atom symbols/order and defines the atom count.
`--vel`	none	Optional fallback initial velocity file if velocities are not found inside the Gaussian output tables.
`-o, --output`	`parsed_log`	Basename for outputs produced by `--parse-dyn`: `<output>_xv.xyz`, optional movie `<output>.xyz`, optional energies `<output>_energies.dat`, optional nimbus trajectory.
`--Emin`	0.0	Reference minimum potential energy in Hartree. Used only if `--scale` is enabled.
`--scale`	off	If enabled, shifts parsed `Epot` and `Etot` by subtracting `--Emin`.
`--movie`	off	Write coordinates-only XYZ (for visualization, e.g. VMD): `<output>.xyz`.
`--total`	off	Write energies file `<output>_energies.dat` containing `EKin/EPot/ETot` per step.
`--nimbus-traj`	off	Also write a flying_nimbus-compatible trajectory: positions in Å and velocities in bohr/au_time.
`--nimbus-out`	none	Explicit output filename for the nimbus trajectory. If omitted, uses `<output>_traj.xyz`.

ORCA/Q-Chem QMD output parsing to flying_nimbus

Option	Default	Meaning / Notes
`--parse-orca-qmd`	off	Parse ORCA MD dumps (`trajectory.xyz` + `velocity.xyz`) and write a flying_nimbus trajectory (x in Å, v in bohr/au_time).
`--parse-qchem-qmd`	off	Parse Q-Chem AIMD/QMD output (positional `input_file`) and write a flying_nimbus trajectory (x in Å, v in a.u.).
`--orca-traj`	`trajectory.xyz`	ORCA positions dump used by `--parse-orca-qmd`.
`--orca-vel`	`velocity.xyz`	ORCA velocities dump used by `--parse-orca-qmd`.
`--epot-out`	none	Optional E_{pot} output file: ORCA: parsed from `trajectory.xyz` comment lines if present; Q-Chem: from `V(Electronic)` if present, else computed as `E(Total)-T(Nuclear)`.

Flying $\nu_i$mbus

Option	Default	Meaning / Notes
`-N, --nat`	required	Number of atoms N.
`--nrototrasl`	6	Number of roto-translational modes N_{rt} to exclude from vibrational analysis. Use 6 for non-linear molecules and 5 for linear molecules. Then $N_{\mathrm{vib}} = 3N - N_{\mathrm{rt}}$.
`--xyz`	required	Equilibrium XYZ used to read element symbols and masses.
`--hess`	required	Hessian (lower triangle after two header lines).
`--traj`	required	Extended XYZ trajectory with velocities.

Option	Default	Meaning / Notes
`--coord`	`nm`	Coordinate representation: `nm` = normal-mode projected spectra, `cart` = Cartesian velocity spectrum/-correlation.
`--modes`	none	List of vibrational mode indices (1-based) for `--coord nm`. Mandatory in NM mode.
`--atoms`	none	Subset of atoms (1-based) for partial spectra. In NM mode this masks Cartesian DOFs before projection (partial contribution to global modes). In Cartesian mode this restricts which atomic velocities contribute.
`--nstart`	1	First trajectory step to use (1-based).
`--ncorr`	2500	Length (number of points) of the analysis window (also the maximum lag in corr-FT).
`--dt`	8.2682749151502	Time step in atomic units.
`--no-ta`	off	Disable TA route and compute correlation functions + FT (corr-FT). By default TA is enabled.
`--nbeads`	0	Number of time origins for correlation averaging. 0 means "auto" (the code uses $N_T - (N-1)$). Used only in corr-FT.
`--nbeadsstep`	1	Stride between time origins. Larger values reduce cost but increase noise. Used only in corr-FT.
`--alpha-pow`	0.0	Gaussian damping parameter α for velocity-like correlation (Cpp). Used only in corr-FT.
`--alpha-dip`	10^{-8}	Gaussian damping parameter for coordinate-like correlation (Cqq). Used only in corr-FT.
`--init-wnumb`	0	Initial wavenumber (cm^{-1}) for output grid.
`--spec-res`	1	Wavenumber spacing $\Delta\tilde{\nu}$ (cm^{-1}).

Option	Default	Meaning / Notes
`--wnumb-span`	5000	Wavenumber span (cm^{-1}). The number of frequency points is $N_f = \lfloor \texttt{wnumb-span}/\texttt{spec-res} \rfloor$.
`--readcnorm`	0	Normal-mode control: 0 = compute normal modes from Hessian and write `cnorm.dat` (will not overwrite), 1 = read `cnorm.dat`.
`--cnorm`	`cnorm.dat`	Path to `cnorm.dat`.
`-o, --output`	`QCT_`	Root output prefix used in filenames.
`--plot`	off	Save PNG plots for each spectrum file.
`--plot-dir`	`.`	Directory for PNG output. PNG name matches the corresponding `.dat` name with `.png` extension.
`--plot-dpi`	200	PNG DPI resolution.
